# Antithrombotic and prohemorrhagic actions of different concentrations of apixaban in patients exposed to single and dual antiplatelet regimens

**DOI:** 10.1038/s41598-023-50347-2

**Published:** 2023-12-27

**Authors:** Julia Martinez-Sanchez, Leticia Castrillo, Didac Jerez, Sergi Torramade-Moix, Marta Palomo, Guiomar Mendieta, M. Urooj Zafar, Ana Belén Moreno-Castaño, Pablo Sanchez, Juan Jose Badimon, Maribel Diaz-Ricart, Gines Escolar, Mercè Roqué

**Affiliations:** 1 Hemostasis and Erythropathology LaboratoryHematopathologyDepartment of Pathology, Centre de Diagnostic Biomedic (CDB), Hospital Clinic de Barcelona, Institut d’Investigacions Biomediques August Pi i Sunyer (IDIBAPS), Universitat de Barcelona, Barcelona, Spain; 2 Josep Carreras Leukaemia Research Institute (Campus Clinic), Barcelona, Spain; 3 Barcelona Endothelium Team, Barcelona, Spain; 4 Department of Cardiology, ICCV, Hospital Clinic de Barcelona, IDIBAPS, Universitat de Barcelona, Villarroel 170, 08036 Barcelona, Spain; 5 Hematology External Quality Assessment Laboratory, CDB, Hospital Clinic de Barcelona, Barcelona, Spain; 6 Department of Medicine, AtheroThrombosis Research Unit (ATRU), Cardiovascular Institute, Icahn School of Medicine at Mount Sinai, New York, USA; 7 Department of Marine Biology and Oceanography, Institut de Ciències del Mar, Spanish National Research Council, Barcelona, Spain

**Keywords:** Cardiology, Thrombosis, Translational research

## Abstract

We evaluated modifications in the hemostatic balance of different concentrations of apixaban (APIX) in 25 healthy donors and 53 patients treated with aspirin (ASA, n = 21), ASA and clopidogrel (ASA + CLOPI, n = 11), or ASA and ticagrelor (ASA + TICA, n = 21). Blood samples from participants were spiked ex vivo with apixaban 0 (APIX0), 40 (APIX40), and 160 ng/mL (APIX160). We assessed the effects of APIX on (1) clot formation, by ROTEM thromboelastometry; (2) thrombin generation primed by platelets; and (3) platelet and fibrin interactions with a thrombogenic surface, in a microfluidic model with circulating blood. APIX caused dose-related prolongations of clotting time with minimal impact on other ROTEM parameters. Thrombin generation was significantly inhibited by APIX160, with ASA + TICA actions showing the strongest inhibition (p < 0.01 vs APIX0). Microfluidic studies showed that APIX160 was more potent at suppressing platelet and fibrin interactions (p < 0.001 vs. APIX0). APIX40 demonstrated a consistent antithrombotic action but with a favorable protective effect on the structural quality of fibrin. APIX potentiated the antithrombotic effects of current antiplatelet regimens. APIX at 40 ng/mL, enhanced the antithrombotic action of single or dual antiplatelet regimens but was more conservative for hemostasis than the 160 ng/mL concentration.

## Introduction

The incidence of atrial fibrillation (AF) and deep vein thrombosis (DVT) increases with age. It is estimated that 2% of the population in the EU and the USA will have a diagnosis of AF, and 80% of these patients will require anticoagulant treatment^1^. Twenty percent of the patients with AF will require revascularization procedures during their lives^2,3^. It is estimated that 5–7% of patients undergoing percutaneous coronary interventions (PCI) will have indications for chronic oral anticoagulant therapy^4^.

Patients undergoing PCI, peripheral artery revascularization, or post-acute coronary syndrome (ACS), require dual antiplatelet therapy (DAPT) consisting of aspirin and a P2Y_12_ receptor antagonist^5^. The recent availability of newer direct oral anticoagulants (DOACs) and more potent antiplatelet agents (i.e., prasugrel, ticagrelor) has further increased the complexity of managing the thrombosis-bleeding balance in these patients^6^. A recent meta-analysis of 4 randomized controlled trials including 42,411 patients on DOACs, of which 33.4% were also on antiplatelet drugs, reported similar efficacy in thromboembolism prevention in patients on DOACs alone compared with those on both treatments (DOACs + antiplatelet). However, higher rates of bleeding were observed in patients exposed to combination therapy^7^.

Thrombin formation, platelet deposition, and fibrin formation play a major role not only in physiological hemostasis but also in the formation of occlusive thrombi. These processes are modulated by local rheologic conditions^8^. At the doses approved for treatment or prevention of thrombotic complications, DOACs significantly inhibit thrombin generation. In addition to being responsible for the conversion of fibrinogen into fibrin, thrombin is the most powerful platelet agonist^9^. There is a need for studies exploring the mechanisms involved in the additive or synergistic prohemorrhagic effects of triple therapies in patients on chronic dual antiplatelet administration and undergoing revascularization procedures.

Previous in vitro studies with circulating human blood have demonstrated that apixaban (APIX), at a concentration compatible with the C_max_ (160 ng/mL) reached in patients with the standard dose for AF (5 mg BID)^10^, reduced both fibrin and platelet depositions preventing thrombus formation^11^. Interestingly, lower concentrations of APIX (corresponding to 40 ng/mL) compatible with levels reached after 2.5 mg b.i.d. did not inhibit fibrin deposition but still showed some antiplatelet effects under dynamic flow conditions. These studies suggest that there is a differential inhibitory effect of DOACs on fibrin formation and platelet aggregation. This dose-dependent effect may have a significant detrimental impact on hemostasis when concomitantly used with strong antiplatelet therapies.

We aimed to evaluate the antithrombotic/prohemorrhagic actions of standard (160 ng/mL) and a lower (40 ng/mL) concentration of APIX in patients receiving antiplatelet therapies. The studies were carried out ex vivo, spiking APIX concentrations into blood samples drawn from healthy individuals and from patients receiving antiplatelet treatments for primary or secondary prevention of ischemic events.

## Results

### Demographics of the study population

**Figure 1:**
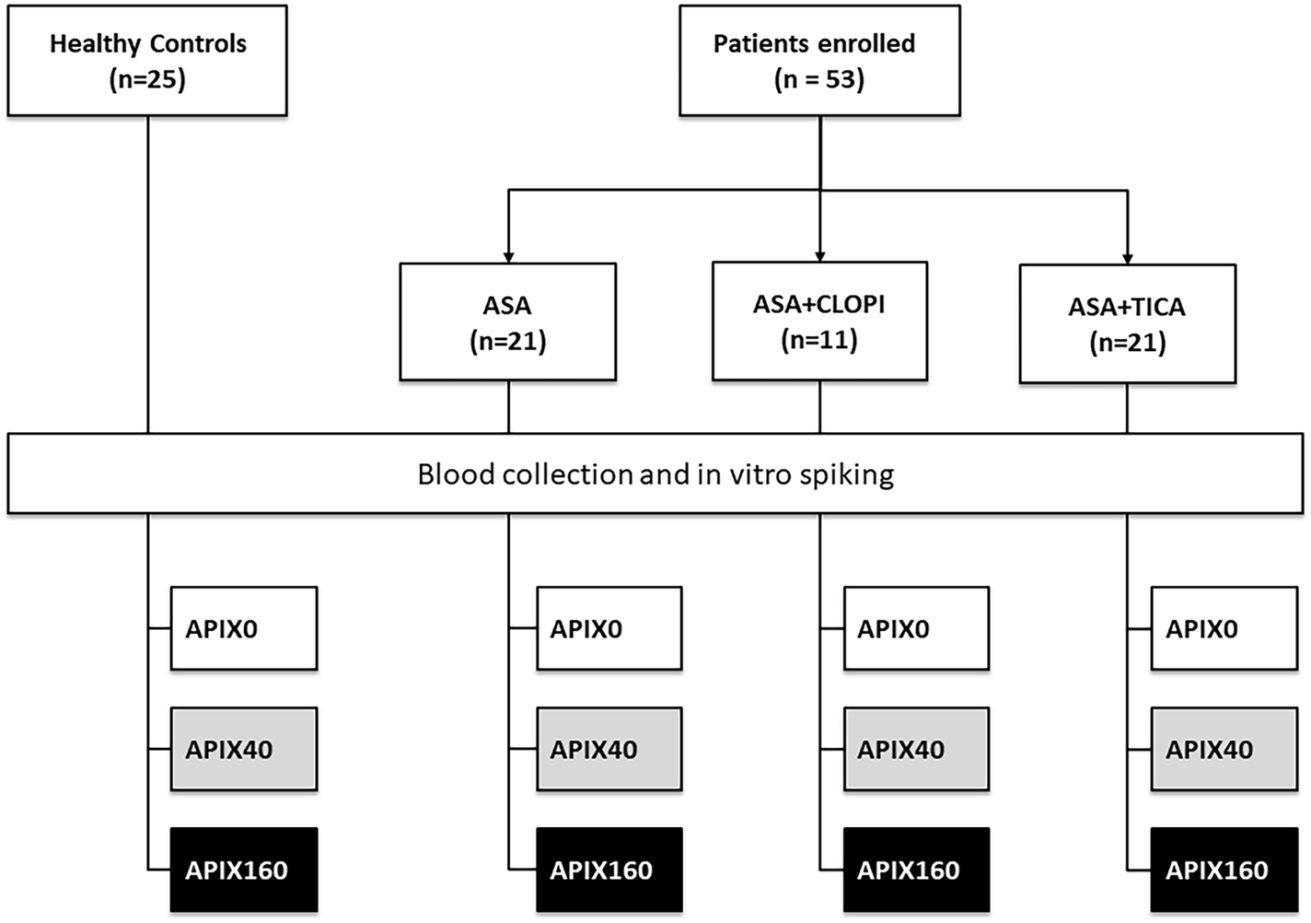
The diagram summarizes the design of the study. APIX was spiked on blood samples from the different study groups: healthy controls, or patients under treatment with aspirin (ASA), aspirin plus clopidogrel (ASA + CLOPI), or aspirin plus ticagrelor (ASA + TICA). APIX was initially solved in dimethyl sulfoxide (DMSO)and further diluted in phosphate-buffered saline (PBS) containing a low concentration of DMSO. Volumes spiked to blood samples for Control or treatment groups were equal (100 µL per 2 mL of blood or equivalent). Concentrations of APIX were corrected for the small volume change. Concentrations of DMSO were kept identical (< 0.05%) in all blood samples.

Figure 1 summarizes the study design and the distribution of control and treated groups Characteristics of patients and control groups are summarized in Table 1. The mean age of the participants was 62 years, 65% of whom were male. The patients in the study had a significant burden of cardiovascular risk factors; 43% were smokers; 52% had a history of hypertension, 24% had diabetes and 43% had dyslipidemia. Data Among all screened patients, 103 patients were finally recruited, and 52 patients completed the full study. Participants in the study were healthy participants not subjected to antithrombotic treatment (Controls), patients on aspirin treatment (ASA), or dual antiplatelet therapy with aspirin + clopidogrel (ASA + CLOPI) or aspirin + ticagrelor (ASA + TICA).

**Table 1:** Demographics and cardiovascular risk factors of the study population.

	Age (mean ± SD)	Male (%)	Smokers (%)	Hypertension (%)	Diabetes (%)	Dyslipidemia (%)
Control n = 25	54 ± 21	50	21	39	14	18
AS n = 21	70 ± 15	59	41	64	23	64
ASA + CLOPI n = 11	68 ± 15	91	64	73	36	73
ASA + TICA n = 21	62 ± 10	76	67	48	33	43
Total n = 78	62 ± 17	65	43	52	24	43

Functional studies on platelet responses in the PFA-200 confirmed the inhibitory effects on platelet functions expected for those patients subjected to antiplatelet treatments. No obvious evidence of high on-treatment platelet reactivity (HPR) was detected in our patient population (Supplementary Material_Table 1).

### Effects of apixaban on thrombus dynamics

**Figure 2:**
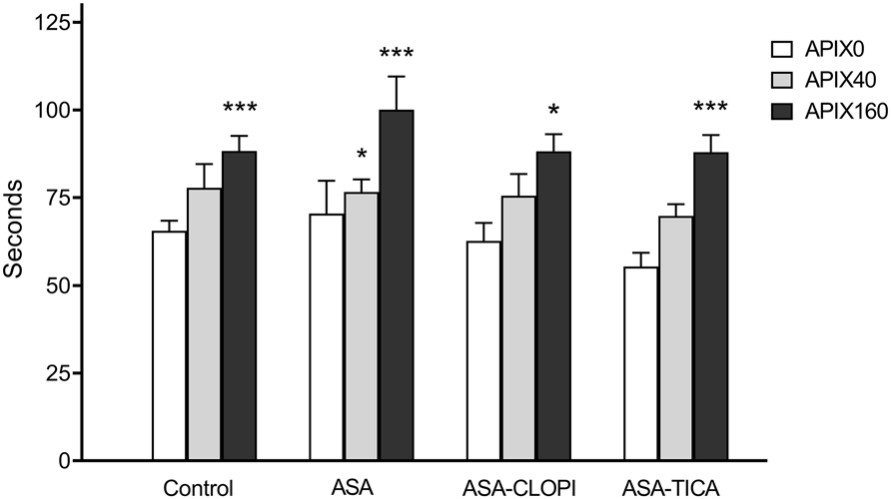
Modifications in clotting times in ROTEM studies. Bars summarize the results of clotting times in seconds (mean ± SEM) for Control and treatment groups. Apixaban caused a dose-related prolongation of clotting times in all study groups. Prolongations in clotting times were more evident with APIX160 and less pronounced in the group exposed to APIX40. *p < 0.05; **p < 0.01; ***p < 0.001; vs. respective APIX0. Control (n = 25); ASA (n = 21) ASA + CLOPI (n = 11), ASA + TICA (n = 21).

As summarized in Fig. 2, viscoelastic parameters triggered by EXTEM, were within the normal ranges in baseline samples (APIX0) from all participants No statistically significant differences were observed for the ROTEM parameters among the different antiplatelet treatment regimes. APIX spiking was associated with dose-related prolongations of CT, with APIX160 showing statistically significant differences for ASA + CLOPI (p < 0.01) and ASA or ASA + TICA treated patients (p < 0.001) versus APIX0. APIX40 also prolonged the CT, but it was statistically significant only for the ASA group (p < 0.05 vs APIX0). Minimal reductions in MCF were observed for APIX40 or r APIX160 in the different groups of patients (refer to Supplementary Material_Table 2 for more detailed information).

### Evaluations of thrombin generation assay

**Figure 3:**
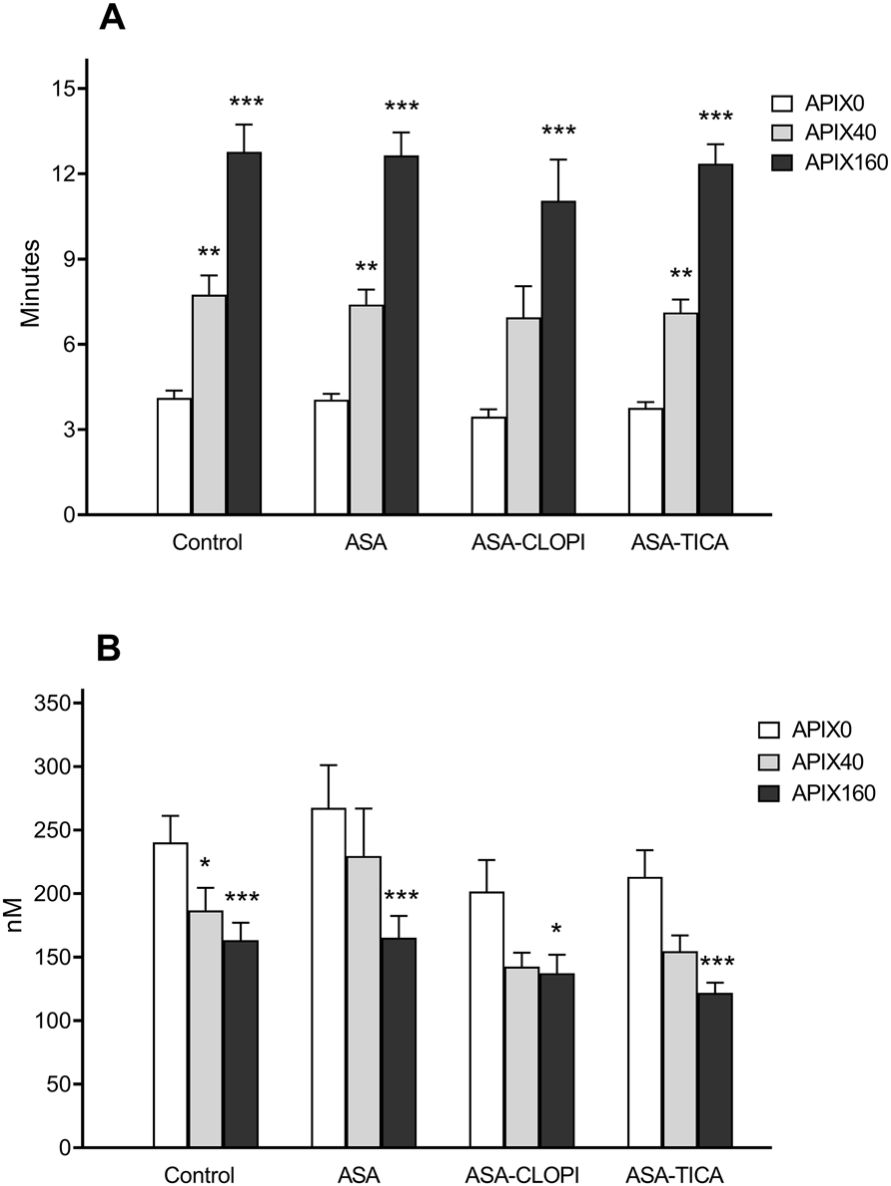
Modifications in lag times (**A**) and thrombin peaks (**B**) in thrombin generation assays. Bars summarize the results expressed as mean ± SEM of lag times (minutes) and thrombin peaks (nM) for Control and treatment groups. Apixaban caused a dose-related prolongation of lag times with a subsequent reduction in thrombin peaks. Prolongations in lag times were superior with APIX160 and less pronounced in the group exposed to APIX40. Reductions in thrombin peaks were more evident with APIX160. *p < 0.05; **p < 0.01; ***p < 0.001; vs. respective APIX0. Control (n = 25); ASA (n = 21) ASA + CLOPI (n = 11), ASA + TICA (n = 21).

Figure 3A and B summarize variations in lag times and maximum thrombin peaks measured in our studies. The addition of APIX at 40 or 160 ng/mL to the healthy-control group resulted in dose-related prolongations of the lag time (p < 0.01 and p < 0.001 respectively vs. APIX0) with a concomitant significant reduction in the thrombin peak. Prolongations in lag times after the in vitro addition of APIX40 or APIX160 to blood from patients treated with ASA, ASA + CLOPI, or ASA + TICA followed similar tendencies to those observed in the Control group. A progressive reduction in maximum thrombin peak was observed when APIX40 or APIX160 were added to samples of blood from patients treated with all the antiplatelet regimens (ASA, ASA + CLOPI, or ASA + TICA). APIX160 caused a marked reduction in thrombin peaks that were more evident in blood from patients treated with ASA and ASA + TICA (p < 0.001 vs APIX0. Times to reach the thrombin peak (time to peak) were consistently associated with prolongations on lag time and reductions in thrombin generation previously commented, but differences did not reach levels of statistical significance (refer to Supplementary Material_Table 3).

**Figure 4:**
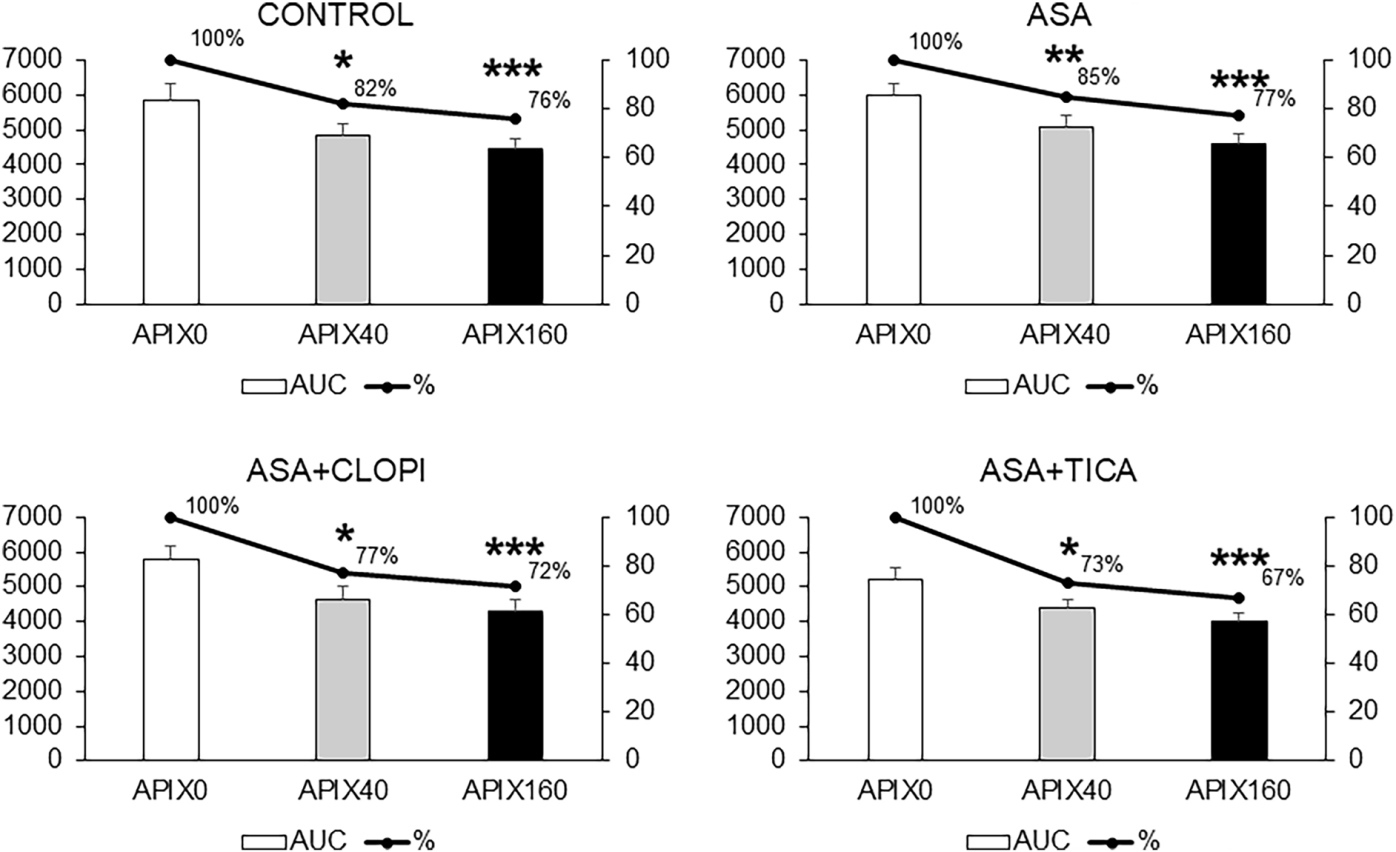
Areas under the curve (AUC) calculated during the thrombin generation assay for the different study groups. Bars summarize the results of total thrombin generation in the different groups. The solid lines provide a visual estimate of the progressive effects calculated as a percentage of the AUC in the absence of apixaban (APIX0). Reductions in total thrombin generation appear more intense in the group of patients exposed to APIX + TICA, less pronounced in the group of patients treated with ASA, and somewhere intermediate in the group of patients treated with ASA + CLOPI. AUC expressed as arbitrary units *p < 0.05; **p < 0.01; ***p < 0.001; vs. respective APIX0. Control (n = 25); ASA (n = 21) ASA + CLOPI (n = 11), ASA + TICA (n = 21).

Figure 4 summarizes the results of the total thrombin generation expressed as area under the curve (AUC in arbitrary units) for the different study groups. Exposure to APIX 40 or 160 ng/mL resulted in progressive reductions in the AUC in healthy subjects and also in the treated groups with the inhibitory effect being always more significant for APIX160 (p < 0.001 vs APIX0). The AUC in control studies (APIX0) was reduced in the presence of APIX 40 or 160 ng/mL (p < 0.05 and p < 0.001 respectively vs. APIX0). AUC for the ASA group was significantly reduced with APIX40 or APIX160 (p < 0.01 and p < 0.001 respectively vs. APIX0). Reductions in AUC were also observed in patients exposed to ASA + CLOPI or ASA + TICA with differences reaching statistical significance at p < 0.05 for APIX40 and p < 0.001 for APIX160 vs. APIX0.

### Effect on platelet and fibrin interactions

**Figure 5:**
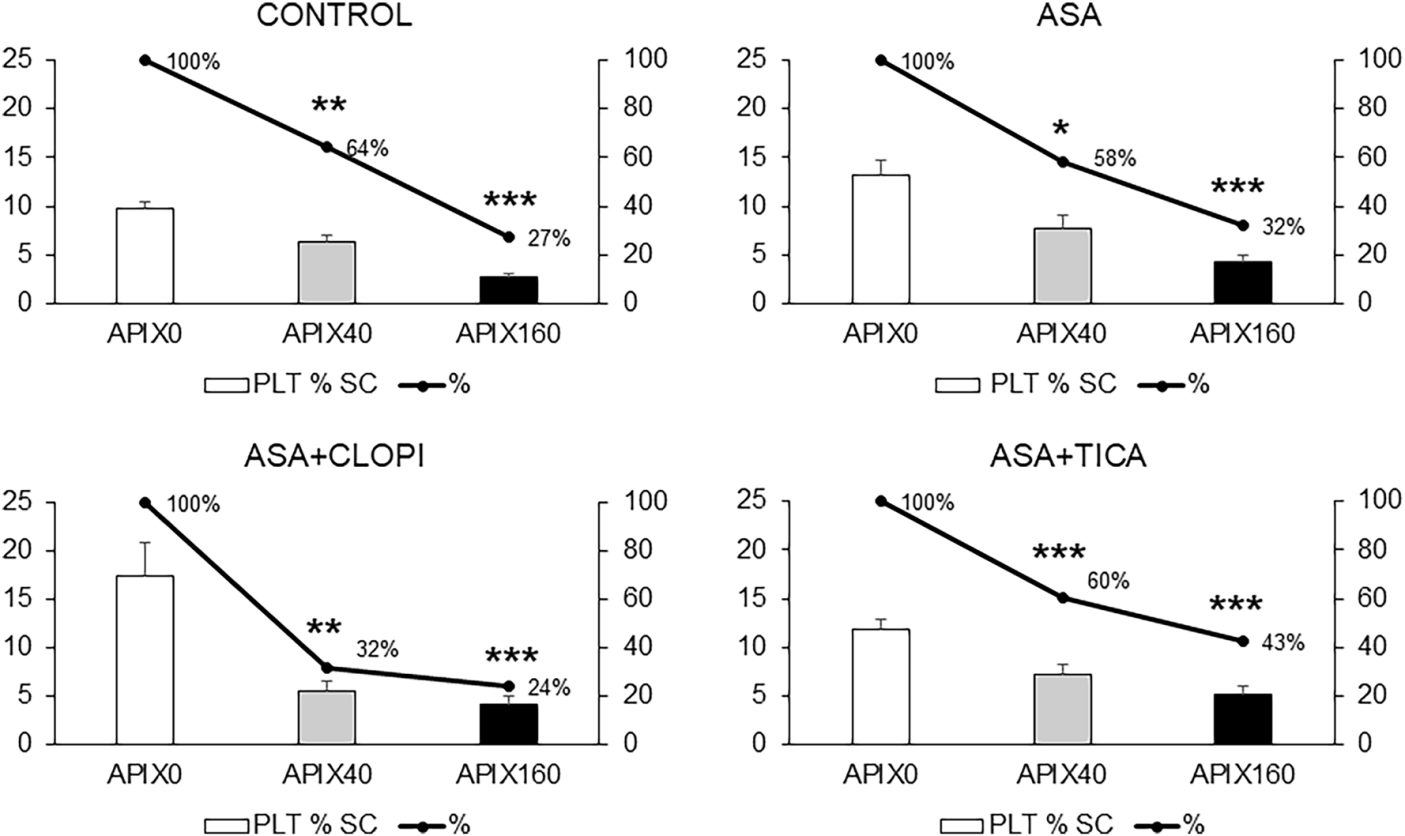
Percentages of the perfused surface covered by platelets. Bars summarize percentages of the surface covered by platelets in microfluidic studies evaluated by confocal microscopy for the different study groups. The solid lines provide a visual estimate of the progressive inhibitory effects calculated as a percentage of the platelet coverage in the absence of apixaban (APIX0). APIX40 significantly reduced the association of platelets to the forming thrombi. Reductions were more significant in studies with APIX160. Reductions in the combination of APIX160 with antiplatelet regimens seem more intense in the group of patients exposed to APIX + TICA, less prominent in the group of patients treated with ASA, and somewhere intermediate in the group of patients treated with ASA + CLOPI. *p < 0.05; **p < 0.01; ***p < 0.001; vs. respective APIX0. Control (n = 9); ASA (n = 6) ASA + CLOPI (n = 4), ASA + TICA (n = 8).

Figure 5 illustrates the inhibitory activity of APIX on platelet and fibrin deposition on the thrombogenic surface of our microfluidic chamber. The inhibition effect was dependent on the apixaban concentrations and more evident with APIX160. The control group showed a platelet surface coverage of 9.7 ± 0.8, 6.3 ± 0.7, and 2.6 ± 0.3% for APIX 0, 40, and 160 ng/mL respectively (p < 0.01 and p < 0.001 respectively vs. APIX0). Of interest, the platelet coverage for the ASA-treated patients was 13.2 ± 1.5, 7.7 ± 1.4, and 4.2 ± 0.6% for APIX 0, 40, and 160 ng/mL (p < 0.05 and p < 0.001 respectively vs. APIX0), respectively. In patients treated with ASA + CLOPI, platelet coverage was 17.4 ± 3.5, 5.6 ± 0.9, and 4.2 ± 0.8%, for APIX 0, 40 or 160 ng/mL (p < 0.01 and p < 0.001 respectively vs. APIX0). In patients treated with ASA + TICA, platelet surface coverages observed were 11.9 ± 1.3; 7.2 ± 0.5, and 5.1 ± 0.5% for APIX 0, 40, and 160 ng/mL (p < 0.001 vs. APIX0 for both concentrations).

**Figure 6:**
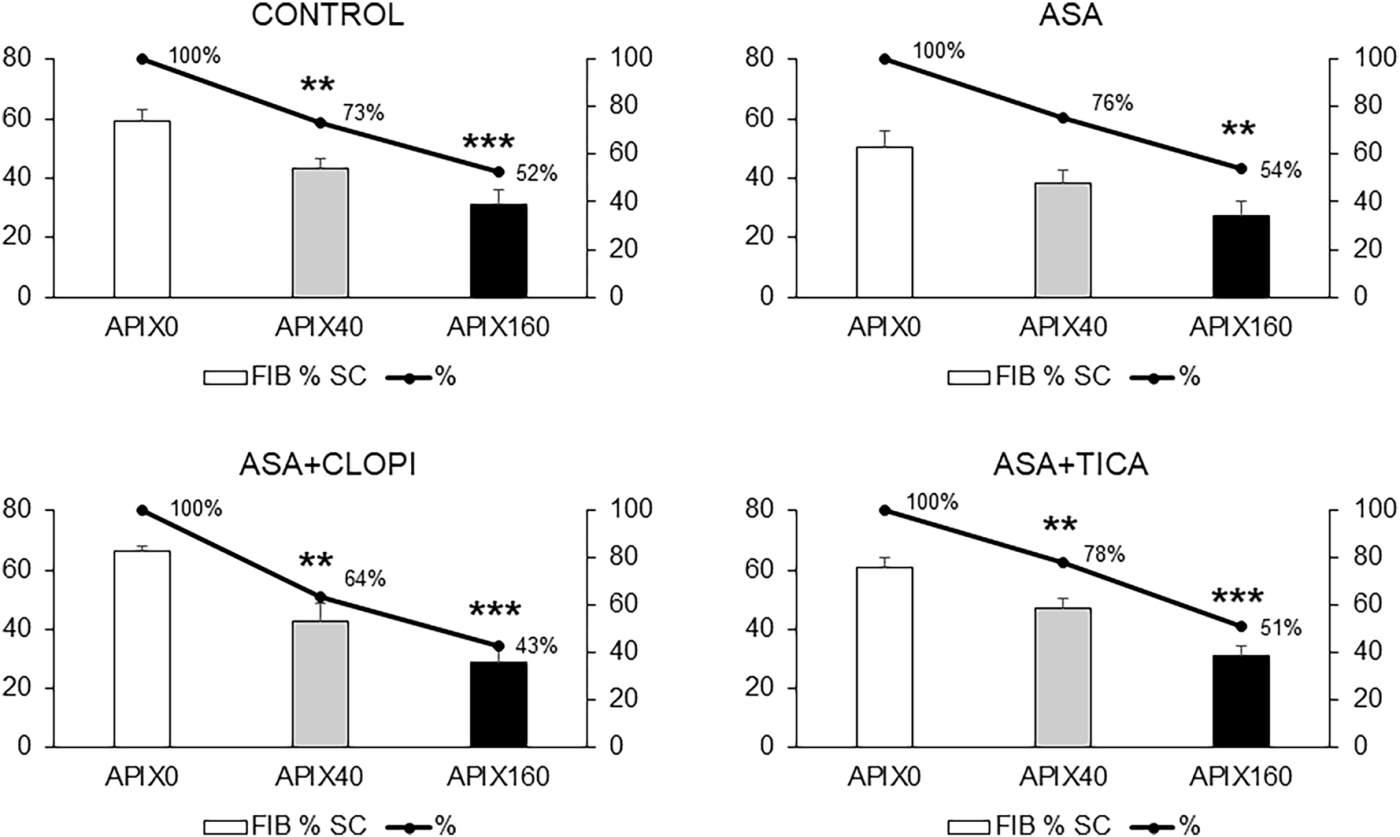
Percentages of the perfused surface covered by fibrin. Bars summarize percentages of the surface covered by fibrin in microfluidic studies evaluated by confocal microscopy for the different study groups. The solid lines provide a visual estimate of the progressive inhibitory effects calculated as a percentage of fibrin coverage in the absence of apixaban (APIX0). APIX40 significantly reduced the association of fibrin to the forming thrombi. Reductions were more significant in studies with APIX160 *p < 0.05; **p < 0.01; ***p < 0.001; vs. respective APIX0. Control (n = 9); ASA (n = 6) ASA + CLOPI (n = 4), ASA + TICA (n = 8).

The effects on fibrin coverage are presented in Fig. 6 showing a similar trend as previously described for platelet coverages. The Control group showed 59.2 ± 3.9, 43.3 ± 3, and 31 ± 4.9% for APIX 0, 40, and 160 ng/mL (p < 0.01 and p < 0.001 respectively vs. APIX0). Fibrin coverages in blood from patients treated with ASA were 50.4 ± 5.5, 38.1 ± 4.6, and 27.2 ± 5.1%, for APIX 0, 40, or 160 ng/mL (p < 0.01 vs. APIX0). The ASA + CLOPI group showed Fibrin coverages of 66.3 ± 1.9, 42.4 ± 6.5, and 28.7 ± 5.1% for APIX 0, 40, and 160 ng/mL (p < 0.01 and p < 0.001 respectively vs. APIX0). In patients treated with ASA + TICA, values were 60.7 ± 3.4, 47.3 ± 2.9, and 31.1 ± 3.1% for APIX 0, 40, and 160 ng/mL (p < 0.01 and p < 0.001 respectively vs. APIX0).

**Figure 7:**
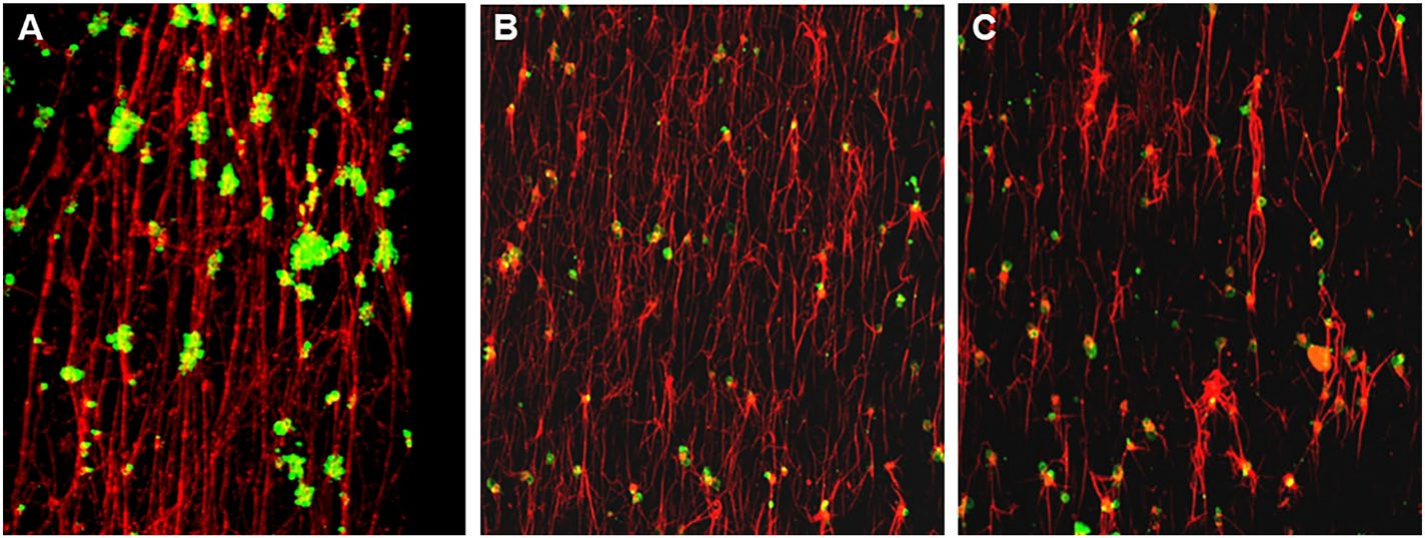
Confocal Images from representative frames from corresponding 3-D image compilations. (**A**) ASA + TICA APIX0, (**B**) ASA + TICA APIX40 and (**C**) ASA + TICA APIX160. Recruitment of platelets was reduced by the antiplatelet agents and further potentiated by the presence of APIX. Structural changes in fibrin fibers and fibrin networks are observed after exposure to APIX40 or APIX160. At the lower concentration (APIX40) apixaban interferes with the thickness and structure of the fibers formed. APIX160 delayed the generation of fibrin and caused more evident disorganization of the fibrin networks formed on thrombogenic surfaces in microfluidic studies. These patterns were consistently observed with the different antiplatelet agents investigated and were maximal for the combination of ASA + TICA.

A detailed evaluation of the confocal images (Fig. 7) and the 3-D projections (Supplementary .MP4 clips) by confocal microscopy, revealed that APIX, in addition to its inhibitory action on fibrin formation, induced changes in the structure of the fibrin networks on the perfused surface. Fibrin fibers were thinner in the presence of APIX40, and their formation was delayed in studies with APIX160.

## Discussion

The results of the present study demonstrate that APIX potentiates the antithrombotic effects of current antiplatelet regimens. The combination of antiplatelet therapy (whether single or dual) with low doses of APIX enhanced the antithrombotic effects of the association, but always with a more conservative profile for hemostasis, with less interference with fibrin formation, than the higher APIX concentrations. The highest APIX dose used in our study corresponds to the plasma levels achieved after the administration of the commonly recommended dose for thromboprophylaxis in patients with atrial fibrillation. These data provide experimental evidence supporting the present recommendations from the clinical guidelines recommending a more conservative (or less intense) alternative/approach for patients at higher bleeding risk and on antiplatelet therapy. The alternative approach would be either to reduce the duration of dual antiplatelet therapy or the dose of anticoagulant to decrease bleeding risk without significantly impacting the antithrombotic efficacy of the DOACs.

A large proportion of patients with atrial fibrillation on chronic DOACs undergoing stent-implantation will also require dual antiplatelet therapy for a certain period. The combination of the anticoagulant treatment due to coexistent atrial fibrillation or deep venous thrombosis with the antiplatelet therapy will entail a higher risk for bleeding complications in this group of patients.

There is limited information from studies exploring the interactions of DOACs with antiplatelet agents^12^. Several clinical studies have explored the safety/efficacy balance of the association of DOACs and antiplatelet therapies with variable results^13^. In an initial study, the APPRAISE-2 trial investigated the beneficial effect of APIX (5 mg BID, the full anticoagulant dose recommended for AF) which resulted in an increased rate of major bleeding events without benefits on the incidence of major cardiovascular events^14^, results which are consistent with those of our mechanistic study.

The PIONEER AF-PCI successfully treated patients with atrial fibrillation undergoing PCI with lower doses of rivaroxaban (2.5 mg twice daily) in combination with dual antiplatelet therapy^15^. The RE-DUAL PCI combined regular doses of dabigatran, adjusted per age and comorbidities, associated with a P2Y_12_ inhibitor, compared with dual antiplatelet therapy plus warfarin^16^. Those previous studies showed comparable efficacy in thromboembolic events prevention and a decreased bleeding risk with DOACs, specifically in intracranial hemorrhage, the most feared bleeding complication. Later on, the AUGUSTUS trial demonstrated APIX superiority compared to anti-vitamin K drugs in the prevention of ischemic cerebral events and a significant reduction of the bleeding risk in the group treated with APIX and the P2Y_12_ inhibitor alone, without aspirin^17^. Recent clinical trials are considering the reduction of APIX to lower doses when concomitant antiplatelet treatment is indicated^18^. Overall evidence from the previously mentioned studies suggests the possibility of balancing the risk/benefit ratio by individualizing doses of DOACs in those clinical conditions requiring dual or triple antithrombotic therapies.

Our results support the validity of an experimental approach similar to the one undertaken by us evaluating platelet and coagulation components of hemostasis can help to select the most adequate antithrombotic treatments for patients receiving both, DOACs and antiplatelet therapy. In summary, our data show that APIX at its standard dosage (5 mg BID), may exhibit synergistic activity with direct P2Y_12_ receptor blockers such as clopidogrel or ticagrelor. Moreover, the results from our study provide additional mechanistic insights into how these antithrombotic agents work in combination and how APIX or other DOACs may reduce the contribution of thrombin to platelet reactivity.

APIX caused an alteration of some viscoelastic parameters as assessed by ROTEM, characterized by a dose-related prolongation of the clotting time (CT) with a surprisingly minimal impact on maximum clot firmness (MCF). These findings confirm the results of previous studies revealing that DOACs prolong clotting time without impacting maximum clot strength in whatever the DOACs or the concentrations used^11,19–24^.

Thrombin generation in samples of plasma from the different groups of patients evaluated in our study revealed more consistent differences among treatment groups. Thrombin generation tests have been used to identify bleeding risks in patients exposed to dual antiplatelet therapy^25^. Results from our present study indicate that the time to initiation of thrombin generation was progressively delayed by the increasing concentrations of APIX. Thrombin peaks and total amounts of thrombin generated seemed to be affected by the potency of the antiplatelet therapies being more important in patients on ASA + TICA than in those on ASA + CLOPI and ASA. These results are in agreement with another study, where thrombin generation assays induced by tissue factor plus ADP in human platelet-rich plasma showed as a potential measurement to assess the effect of the concomitant use of an oral factor Xa inhibitor and P2Y_12_ receptor antagonists^26^. Although results of thrombin generation assays do not follow a strict correlation with levels of apixaban in treated patients^27^ these assays provide more accurate information on the dynamics of thrombin generation that is not offered by the viscoelastic technologies in ROTEM.

Confocal analysis of microfluidic studies revealed a progressive effect of the anticoagulant APIX and antiplatelet therapies. APIX reduced platelet and fibrin interactions with the thrombogenic surface exposed to circulating blood. Inhibition in platelets and fibrin formation has been reported for different DOACs in previous studies from our group and others^20,21,28,29^. Interestingly, one of the previous studies reported a delayed fibrin formation in DOAC-treated under static conditions^29^. Our present microfluidic studies with recalcified anticoagulated blood were performed for a relatively short time (3 min) to avoid clotting of the system. Thus, these conditions can only explore the initiation of a hemostatic plug. Results demonstrate that the presence of APIX significantly impaired both, the formation of fibrin and the recruitment of platelets into forming thrombi. APIX 160 ng/mL was more potent at suppressing platelet and fibrin interactions with the collagen-tissue factor substrata with all antiplatelet treatments, while APIX 40 ng/mL demonstrated a consistent antithrombotic action, but proved more respectful at preserving hemostatic parameters with all antiplatelet regimens.

Structural changes in fibrin fibers and fibrin network density have been associated with alterations in hemostasis. Gauer and cols^30^, reported that anticoagulants interfered with the fibrin clot structure, most likely through a reduction of thrombin generation. Alterations in the formation and stability of fibrin structures reported in patients with hemophilia can be effectively corrected by pro-hemostatic strategies^31,32^. Our present data indicate that APIX in addition to its inhibitory action on total fibrin formation may also interfere with structural patterns of fibrin networks formed on thrombogenic surfaces. A delayed formation with additional disorganization of fibrin networks found in microfluidic studies was always more evident with the highest concentration of APIX. Overall, reduced incorporation of platelets and delayed fibrin generation intensified by synergistic antithrombotic therapies would result in the formation of a less occlusive hemostatic plug that will likely translate into an enhanced hemorrhagic risk in patients under dual or triple antithrombotic therapies.

Our study has limitations. Individuals in the control group were in general healthier than those receiving antiplatelet treatments. As a real-life study, the antiplatelet regimes described here reflect the genuineness of antithrombotic treatment at our institution. Clinical protocols and guidelines have evolved during the development of our studies. Changes in clinical and therapeutic approaches have had an impact on the conformation and number of patients finally included in the different study groups. Another limitation of our study is not having studied the combined effect of APIX with single P2Y_12_ inhibitors, to reproduce conditions of clinical trials that have studied the combination of DOACs or anti-vitamin K with these agents. Occasional discrepancies of n values in tables with numbers of patients per group are related to technical issues with occasional tests. Confocal analysis of microfluidic studies was not performed in the full patient population due to difficulties in blood sampling volumes and timing.

Overall, our present data indicate that APIX potentiates the antiplatelet effects of current antiplatelet treatments investigated in our studies. The concentration of 40 ng/mL shows a significant antithrombotic action in combination with the different antiplatelet regimens but appears to be more conservative for hemostasis than the concentrations of 160 ng/mL. The latest concentration more closely assimilates to the C_max_ reached after the standard 5 mg BID, recommended for thromboprophylaxis in patients with atrial fibrillation. The 40 ng/mL would be compatible with the C_min_ after the same treatment regimen, (10) but also with the C_max_ achieved after 2.5 mg twice daily approved as an antithrombotic treatment for subgroups of older patients and/or renal impairment^33^.

In summary, the experimental strategy used in our ex vivo setting may provide information on the intensity of the alterations of hemostasis by antithrombotic therapies before designing expensive clinical trials, thus avoiding the exposure of patients to hazardous combinations of antiplatelet and anticoagulant treatments. Newer anticoagulants and antiplatelet agents are being developed and the effects of their combined use will remain a critical problem. In vitro approaches such as the one described in our studies will be useful as a screening method for possible synergistic antithrombotic actions that could severely impair hemostasis in patients.

## Methods

### Study design

This exploratory clinical study was conducted on patients on single or dual antiplatelet therapies, prescribed by their cardiologists for pre-established conditions. All participants provided written informed consent before any study procedures. The study protocol was approved by the Ethics Committee of the Hospital Clinic de Barcelona (CV185-700) and the study complied with all local regulations and the ethical principles of the current Declaration of Helsinki.

Inclusion criteria were age > 18 years and receiving stable antiplatelet therapy for at least one month. The attending physician was responsible for the indication of the antiplatelet therapy to patients with a diagnosis of any cardiovascular condition requiring platelet aggregation inhibition. Exclusion criteria were: renal failure with GFR < 50 mL/min, anemia with hemoglobin levels < 13 g/dL, platelet counts < 140,000/mL or > 400,000/mL, previously known hemostasis disorders, current use of anticoagulants or any drugs potentially interfering with hemostasis (non-steroidal anti-inflammatory drugs and serotonin-reuptake inhibitors) or previous thromboembolic events of unknown etiology.

The study recruitment involved the identification of potential participants from consecutive patients admitted to the Cardiology Department with ACS or heart failure or needing structural procedures (TAVR, percutaneous mitral or tricuspid valve repair). Those requiring antiplatelet therapy were identified as potential qualifiers. Once the minimum period of 4 weeks of treatment stabilization was achieved, they were approached and enrolled for the study upon positive consent.

Figure 1 summarizes the study design and the distribution of control and treated groups. After screening, blood samples were collected from patients at the Cardiology Clinic during a follow-up visit. A group of healthy donors was used as a control. Apixaban was generously provided by Bristol Myers Squibb (Madrid, Spain). Following the manufacturer's advice, a stock solution was initially prepared in DMSO. This stock solution was further diluted in phosphate buffer saline (PBS) with a lower proportion of DMSO to reach the expected concentrations in blood. Volumes spiked to blood samples for control or treatment groups were equal (50 µL/mL of blood). Concentrations of APIX were corrected for the small volume change. Concentrations of DMSO were kept identical (< 0.05%) in all blood samples including controls. Blood samples were spiked with APIX at concentrations equivalent to 0 (APIX0), 40 (APIX40), and 160 ng/mL (APIX160) and incubated for 30 min. Patients or controls were never exposed to the effects of the DOACs.

The antithrombotic effects of the different doses of APIX were assessed on (1) clot formation, by thromboelastometry, using the ROTEM; (2) thrombin generation, applying a cell-based model of coagulation primed by platelets; and (3) platelet and fibrin interactions/depositions onto a thrombogenic substratum (Type I collagen + tissue factor) experimental model of thrombosis, with blood circulating at arterial shear-rates.

### Screening procedures

All participants underwent screening including medical history, physical examination, routine blood works, drugs of abuse testing, EKG, and cardiac imaging techniques when appropriate. The main diagnoses were: ischemic heart disease, elective percutaneous coronary intervention, arrhythmia, heart failure, peripheral arterial revascularization, or ambulatory primary prevention. All concomitant medications taken by the patients were recorded to confirm adherence to the inclusion criteria. Patients were instructed to take their antiplatelet treatment 3–4 h before their study visit.

### Blood collection and functional evaluation of antiplatelet treatments

Citrated blood samples (20 mL) were drawn in 129 M citrate tubes BD Vacutainer™ (ref 3653079) by a clean venipuncture from the study participants. All participants were tested for proper inhibitory response to the antiplatelet treatment they were prescribed. Platelet hemostatic performance in control individuals and patients was evaluated to verify that patients were adequately following or responding to their antiplatelet treatment^34,35^. For this purpose, aliquots of citrated blood were tested in the Platelet Function Analyzer (PFA-200 System, Siemens Healthineers, Barcelona Spain) using Col-Epi, Col-ADP, and P2Y (Innovance^®^) cartridges. Closure times were recorded. Cut-off values for closure times for the different cartridges were Col-Epi > 137 “(aspirin); Col-ADP < 105”; “Innovance P2Y > 106” (for clopidogrel or ticagrelor)^36^.

### Assessment of the anticoagulant action of apixaban in thromboelastometry studies

We evaluated the inhibitory effects of APIX on the dynamics of whole blood coagulation, using the ROTEM Thromboelastometry Analyzer (PentapharmGmbH, München, Germany)^37^. Citrated blood was recalcified with the starTEM reagent and activated with the r-EXTEM reagent (Biometa, Spain) containing tissue factor as the clotting activator. Dynamics of clot formation were followed for 45 min. Coagulation time (CT), the time (sec) elapsed from the start until the amplitude of the forming clot reaches 2 mm, and maximum clot firmness (MCF) the maximum amplitude of the tracing reached (in mm), were assessed in our ROTEM studies.

### Evaluation of thrombin generation

The contribution of platelets to local thrombin generation was evaluated in a cell-based model of thrombin generation primed by platelets, using a fluorogenic assay (Technoclone GmBH, Austria) as previously described^11^. Briefly, this cell-based model consists of isolated platelets at 1 × 10^6^ platelets/µL from controls or treated blood samples resuspended in Hanks' balanced salt solution, with platelets or plasma always from the same participant. Thrombin generation was initiated by the addition of 1.1 pM tissue factor exposed on phospholipids micelles (Thromborel^®^S, Dade Behring, Marburg GmbH, Germany), and a fluorogenic substrate that also contains CaCl_2_ to favor the activation of coagulation mechanisms. The fluorescence generated was evaluated at a wavelength of 390 nm/450 nm (excitation/emission) for 90 min (at intervals of 1 min) and fluorescence units were analyzed with the Thermo Fluoroskan Ascent Software (Technoclone GmbH). Parameters assessed in our studies were lag time (min), maximum thrombin peak (nM), time to achieve this peak (min), and the total amount of thrombin generated as the area under the curve (arbitrary units).

### Measurement of platelets and fibrin deposition in microfluidic studies

Microfluidic studies were carried out using commercially available slide-based chambers (μ-SlideVI 0.4, IBIDI) coated with fibrillar collagen type I and tissue factor (TF)^38^. Aliquots of citrated whole blood were incubated with APIX concentrations calculated to give rise to concentrations 0 (APIX0), 40 (APIX40), and 160 ng/mL (APIX160) for 30 min, then recalcified with a CaCl_2_ solution, and immediately perfused through the slide micro-channels at a shear rate of 600 s^−1^, for 3 min. Perfused channels were rinsed with 0.15 M PBS, fixed with paraformaldehyde 1% for 15 min at 4 ºC, and further incubated with glycine 1% for 10 min to reduce high background staining due to free unreactive aldehyde groups. Thereafter, perfused channels were exposed to 1% bovine serum albumin (BSA) for 15 min before incubation with specific antibodies to discriminate the contribution of platelets and fibrin, a combination of indirect and direct immunofluorescence. First, platelets were labeled using a mouse anti-CD36 primary antibody for 1 h at RT in a humidified chamber. Then, a secondary antibody anti-mouse Alexa fluor 488 was incubated together with a conjugated antibody anti-fibrinogen Alexa fluor 594 to stain fibrin deposition for 1 h at RT in a humidified chamber.

### Evaluation of platelets and fibrin deposition using confocal microscopy

Fibrin and platelet interactions with the collagen-TF surface were evaluated using confocal microscopy^38^. Perfused channels were observed on a Leica TCS-SP5 Laser Scanning Confocal Microscope. Alexa fluor 488 (green) and Alexa Fluor 594 (red) and reflection images were acquired sequentially using 488 and 561 nm laser lines, AOBS (Acoustic Optical Beam Splitter) as a beam splitter, and emissions from the different channels were acquired through internal photomultipliers at the emission ranges of 500–550 nm, 571–625 nm, 555–565 nm, for reflection, respectively^39^. The confocal pinhole was set at 1 Airy unit. Transmitted light bright field images were acquired simultaneously through a transmitted light detector. The percentage of the surface covered by platelets and fibrin was quantified using Image-J software^40^.

### Statistics

Results are expressed as mean ± standard error of the mean (SEM). Comparisons between different concentrations of APIX vs. baseline values or the control healthy groups were evaluated. ANOVA and Friedman’s or Kruskall–Wallis tests with Dunn’s correction for multiple comparisons were used for statistical comparisons before and after exposure to APIX treatment. The minimal level of statistical significance was set at p < 0.05.

### Ethics approval

The study protocol was approved by the Ethics Committee (CV185-700) of our Institution and the study complied with all local regulations and the ethical principles of the current Declaration of Helsinki.

### Consent to participate

Informed consent to participate in the study was obtained from all individual participants included.

### Supplementary Information

Supplementary Table 1.

Supplementary Table 2.

Supplementary Table 3.

Supplementary Video 1.

Supplementary Video 2.

Supplementary Video 3.

## Data Availability

All authors agree that all data and materials support the published claims and comply with field standards.

## Abbreviations

APIX: Apixaban
APIX0: Apixaban 0 ng/mL
APIX40: Apixaban 40 ng/mL
APIX160: Apixaban 160 ng/mL
ASA: Aspirin (acetyl salicylic acid)
ASA + CLOPI: Aspirin + clopidogrel
ASA + TICA: Aspirin + ticagrelor
CLOPI: Clopidogrel
MCF: Maximum clot firmness
PFA: Platelet function analyzer
ROTEM: Rotational thromboelastometry
TICA: Ticagrelor
VASP: Vasodilator-stimulated phosphoprotein

## Code availability

The authors affirm that software application or custom code is not applicable in this study.
