# Supplementary material for: Antithrombotic and prohemorrhagic actions of different concentrations of apixaban in patients exposed to single and dual antiplatelet regimens
Source: Sci Rep. 2023 Dec 27;13:22969. doi: 10.1038/s41598-023-50347-2 (PMC10752876; doi:10.1038/s41598-023-50347-2)
Supplement: Supplementary file 1 — Supplementary Table 1. [file 41598_2023_50347_MOESM1_ESM.docx]

**SUPPLEMENTARY TABLE 1: PFA-200**

**Supplementary Table 1** Closure times (sec.) in the Platelet Function Analyzer for the different cartridges

|  | **PFA-200 sec.** | | | | | | | | | | | |
| --- | --- | --- | --- | --- | --- | --- | --- | --- | --- | --- | --- | --- |
|  | **CONTROL** | | | **ASA** | | | **ASA+CLOPI** | | | **ASA+TICA** | | |
|  | **COL-ADP** | **COL-EPI** | **P2Y** | **COL-ADP** | **COL-EPI** | **P2Y** | **COL-ADP** | **COL-EPI** | **P2Y** | **COL-ADP** | **COL-EPI** | **P2Y** |
| N | 22 | 22 | 21 | 20 | 22 | 19 | 11 | 11 | 11 | 21 | 21 | 21 |
| Mean | 96.91 | 112.27 | 82.38 | 109.65 | 170.09 **↑** | 95.89 | 216.82 **↑** | 184.91 **↑** | 255.55 **↑** | 263.76 **↑** | 261.14 **↑** | 290.24 **↑** |
| SEM | 4.85 | 6.89 | 7.53 | 7.97 | 18.60 | 8.60 | 26.97 | 28.04 | 25.13 | 15.29 | 14.48 | 5.83 |

Normal Range:

COL-ADP 90-105 sec

COL-EPI 100-120 sec

P2Y <106 sec

↑ = Prolonged
