# Supplementary material for: Antithrombotic and prohemorrhagic actions of different concentrations of apixaban in patients exposed to single and dual antiplatelet regimens
Source: Sci Rep. 2023 Dec 27;13:22969. doi: 10.1038/s41598-023-50347-2 (PMC10752876; doi:10.1038/s41598-023-50347-2)
Supplement: Supplementary file 2 — Supplementary Table 2. [file 41598_2023_50347_MOESM2_ESM.docx]

**SUPPLEMENTARY TABLE 2: ROTEM**

**Supplementary Table 2** Modifications in the most relevant thromboelastometric parameters by apixaban for the different study groups

|  | **CLOTTING TIME (CT) sec.** | | | | | | | | | | | |
| --- | --- | --- | --- | --- | --- | --- | --- | --- | --- | --- | --- | --- |
|  | **CONTROL** | | | **ASA** | | | **ASA+CLOPI** | | | **ASA+TICA** | | |
|  | **APIX0** | **APIX40** | **APIX160** | **APIX0** | **APIX40** | **APIX160** | **APIX0** | **APIX40** | **APIX160** | **APIX0** | **APIX40** | **APIX160** |
| N | 25 | 25 | 25 | 21 | 21 | 21 | 10 | 11 | 11 | 21 | 21 | 21 |
| Mean | 65.52 | 77.76 | 88.28 | 70.43 | 76.57 | 100.10 | 62.60 | 73.18 | 85.45 | 55.43 | 69.76 | 87.95 |
| SD | 14.35 | 33.99 | 21.73 | 42.93 | 16.60 | 43.31 | 16.37 | 20.26 | 17.22 | 17.47 | 15.30 | 22.51 |
| SEM | 2.87 | 6.80 | 4.35 | 9.37 | 3.62 | 9.45 | 5.18 | 6.11 | 5.19 | 3.81 | 3.34 | 4.91 |
| p vs APIX0 |  | 0.472 | 0.000 |  | 0.026 | 0.000 |  | 0.438 | 0.030 |  | 0.076 | 0.000 |
| * | N.S. |  | *** |  | * | *** |  |  | * |  |  | *** |
|  |  |  |  |  |  |  |  |  |  |  |  |  |
|  | **MAXIMUM CLOT FIRMNESS (MCF) mm** | | | | | | | | | | | |
|  | **CONTROL** | | | **ASA** | | | **ASA+CLOPI** | | | **ASA+TICA** | | |
|  | **APIX0** | **APIX40** | **APIX160** | **APIX0** | **APIX40** | **APIX160** | **APIX0** | **APIX40** | **APIX160** | **APIX0** | **APIX40** | **APIX160** |
| N | 25 | 25 | 25 | 21 | 21 | 21 | 10 | 11 | 11 | 21 | 21 | 20 |
| Mean | 65.76 | 63.00 | 64.8 | 68.14 | 63.71 | 64.71 | 64.90 | 58.55 | 62.36 | 69.14 | 68.14 | 65.90 |
| SD | 6.73 | 7.46 | 6.55 | 6.06 | 7.44 | 7.65 | 5.26 | 7.63 | 7.09 | 4.69 | 6.29 | 6.39 |
| SEM | 1.35 | 1.49 | 1.31 | 1.32 | 1.62 | 1.67 | 1.66 | 2.30 | 2.14 | 1.02 | 1.37 | 1.43 |
| p vs APIX 0 |  | 0.102 | 0.999 |  | 0.001 | 0.135 |  | 0.030 | 0.7907 |  | 0.999 | 0.027 |
| * | N.S. |  |  |  | ** |  |  | * |  |  |  | * |

*p<0.05; **p<0.01; ***p<0.001; vs. respective APIX0 using Friedman test with Dunn’s correction for multiple comparisons

N.S. No significant differences among values for APIX0 and the treated cohorts, using the Kruskall-Wallis test with Dunn´s correction for multiple comparisons
