# Supplementary material for: Antithrombotic and prohemorrhagic actions of different concentrations of apixaban in patients exposed to single and dual antiplatelet regimens
Source: Sci Rep. 2023 Dec 27;13:22969. doi: 10.1038/s41598-023-50347-2 (PMC10752876; doi:10.1038/s41598-023-50347-2)
Supplement: Supplementary file 3 — Supplementary Table 3. [file 41598_2023_50347_MOESM3_ESM.docx]

**SUPPLEMENTARY TABLE 3: TGA**

**Supplementary Table 3** Summary of the results obtained on the most relevant parameters of the thrombin generation assay for the different study groups

|  | **LAG TIME min** | | | | | | | | | | | |
| --- | --- | --- | --- | --- | --- | --- | --- | --- | --- | --- | --- | --- |
|  | **CONTROL** | | | **ASA** | | | **ASA+CLOPI** | | | **ASA+TICA** | | |
|  | **APIX0** | **APIX40** | **APIX160** | **APIX0** | **APIX40** | **APIX160** | **APIX0** | **APIX40** | **APIX160** | **APIX0** | **APIX40** | **APIX160** |
| N | 22 | 22 | 22 | 20 | 20 | 20 | 10 | 10 | 10 | 21 | 21 | 21 |
| Mean | 4.11 | 7.75 | 12.77 | 4.05 | 7.40 | 12.65 | 3.45 | 6.95 | 11.05 | 3.76 | 7.12 | 12.36 |
| SD | 1.19 | 3.17 | 4.50 | 0.92 | 2.36 | 3.60 | 0.83 | 3.47 | 4.59 | 0.93 | 2.09 | 3.12 |
| SEM | 0.36 | 0.96 | 1.36 | 0.20 | 0.53 | 0.81 | 0.26 | 1.10 | 1.45 | 0.20 | 0.46 | 0.68 |
| p vs APIX0 |  | 0.005 | 0.000 |  | 0.005 | 0.000 |  | 0.076 | 0.000 |  | 0.004 | 0.000 |
| * | N.S. | ** | *** |  | ** | *** |  |  | *** |  | ** | *** |
|  |  |  |  |  |  |  |  |  |  |  |  |  |
|  | **THROMBIN PEAK nM** | | | | | | | | | | | |
|  | **CONTROL** | | | **ASA** | | | **ASA+CLOPI** | | | **ASA+TICA** | | |
|  | **APIX0** | **APIX40** | **APIX160** | **APIX0** | **APIX40** | **APIX160** | **APIX0** | **APIX40** | **APIX160** | **APIX0** | **APIX40** | **APIX160** |
| N | 23 | 23 | 23 | 20 | 20 | 20 | 10 | 10 | 10 | 21 | 21 | 21 |
| Mean | 240.33 | 186.93 | 163.39 | 267.50 | 229.64 | 165.34 | 201.66 | 142.54 | 137.40 | 213.22 | 154.71 | 121.88 |
| SD | 100.67 | 84.94 | 66.14 | 150.28 | 166.85 | 76.71 | 78.35 | 34.77 | 45.99 | 96.15 | 56.72 | 37.04 |
| SEM | 20.99 | 17.71 | 13.79 | 33.60 | 37.31 | 17.15 | 24.78 | 10.99 | 14.54 | 20.98 | 12.38 | 8.08 |
| p vs APIX0 |  | 0.015 | 0.000 |  | 0.120 | 0.000 |  | 0.076 | 0.042 |  | 0.092 | 0.000 |
| * | N.S. | * | *** |  |  | *** |  |  | * |  |  | *** |
|  |  |  |  |  |  |  |  |  |  |  |  |  |
|  | **TIME TO PEAK min** | | | | | | | | | | | |
|  | **CONTROL** | | | **ASA** | | | **ASA+CLOPI** | | | **ASA+TICA** | | |
|  | **APIX0** | **APIX40** | **APIX160** | **APIX0** | **APIX40** | **APIX160** | **APIX0** | **APIX40** | **APIX160** | **APIX0** | **APIX40** | **APIX160** |
| N | 23 | 23 | 23 | 20 | 20 | 20 | 10 | 10 | 10 | 21 | 21 | 21 |
| Mean | 18.74 | 18.04 | 22.11 | 16.25 | 20.23 | 19.10 | 14.80 | 16.90 | 19.80 | 18.76 | 16.90 | 20.88 |
| SD | 15.37 | 7.37 | 9.24 | 8.05 | 9.95 | 3.30 | 10.65 | 4.10 | 2.64 | 10.37 | 4.71 | 5.64 |
| SEM | 3.20 | 1.54 | 1.93 | 1.80 | 2.22 | 0.74 | 3.37 | 1.30 | 0.83 | 2.26 | 1.03 | 1.23 |
| p vs APIX0 |  | 0.999 | 0.315 |  | 0.707 | 0.707 |  | 0.999 | 0.076 |  | 0.228 | 0.369 |
| * | N.S. |  |  |  |  |  |  |  |  |  |  |  |
|  |  |  |  |  |  |  |  |  |  |  |  |  |
|  | **AUC (Area under curve)** | | | | | | | | | | | |
|  | **CONTROL** | | | **ASA** | | | **ASA+CLOPI** | | | **ASA+TICA** | | |
|  | **APIX0** | **APIX40** | **APIX160** | **APIX0** | **APIX40** | **APIX160** | **APIX0** | **APIX40** | **APIX160** | **APIX0** | **APIX40** | **APIX160** |
| N | 21 | 21 | 21 | 20 | 20 | 20 | 10 | 10 | 10 | 21 | 21 | 21 |
| Mean | 5853.49 | 4821.91 | 4466.58 | 5977.81 | 5071.51 | 4612.34 | 5794.99 | 4623.64 | 4286.46 | 5219.59 | 4379.25 | 3998.58 |
| SD | 2085.85 | 1527.85 | 1350.42 | 1594.82 | 1516.09 | 1176.77 | 1215.43 | 1266.41 | 1067.76 | 1471.08 | 1138.75 | 1201.27 |
| SEM | 455.17 | 333.40 | 294.69 | 356.61 | 339.01 | 263.13 | 384.35 | 400.47 | 337.66 | 321.02 | 248.49 | 262.14 |
| p vs APIX0 |  | 0.013 | 0.000 |  | 0.005 | 0.000 |  | 0.042 | 0.000 |  | 0.041 | 0.000 |
| * | N.S. | * | *** |  | ** | *** |  | * | *** |  | * | *** |

*p<0.05; **p<0.01; ***p<0.001; vs. respective APIX0 using Friedman test with Dunn’s correction for multiple comparisons
N.S. No significant differences among values for APIX0 and the other treated cohorts, using the Kruskall-Wallis test with Dunn´s correction for multiple comparisons
